# Opening the “black box” of organizational coaching for implementation

**DOI:** 10.1186/s12913-022-08948-6

**Published:** 2023-02-01

**Authors:** Kathryn Fleddermann, Nora Jacobson, Julie Horst, Lynn M Madden, Eric Haram, Todd Molfenter

**Affiliations:** 1 Department of Industrial and Systems Engineering, University of Wisconsin–Madison, 1513 University Ave, 53706 Madison, WI USA; 2 Institute for Clinical and Translational Research, School of Nursing, University of Wisconsin–Madison, 53705 Madison, WI USA; 3 APT Foundation, 1 Long Wharf Drive, Suite 321, New Haven, CT USA; 4 Haram Consulting, LLC, 413 River Road, ME 04008 Bowdoinham, United States

**Keywords:** Organizational coaching, MOUD, Implementation, NIATx, Substance use

## Abstract

**Background:**

Organizational coaching to promote the implementation of evidence-informed interventions is becoming more popular in healthcare organizations. In order to open the “black box” of coaching for implementation, we first developed, then tested the rigor and utility of a model of coaching for implementation.

**Methods:**

Interviews with nine experienced coaches were conducted and inductively coded to develop a model of coaching for implementation. Later, forty coaching calls with behavioral health organizations in Ohio, Wisconsin, and Florida were analyzed with directed content analysis using a priori codes based on this model.

**Results:**

The coaching work that occurred during these calls aligned closely with the model of coaching for implementation developed by our team. Most coaching work was devoted to building capacity; almost as much work focused on building relationships. Very little coaching work was dedicated to building sustainability. Use of tools for organizational change and implementation remained relatively consistent across all coaching periods.

**Conclusion:**

Understanding what occurs during a successful coaching intervention will improve the effectiveness of coaching as an implementation strategy. Future research should focus on which processes and patterns make coaching more likely to promote specific implementation outcomes.

## Background

The use of coaching is common in many fields, including athletics, business, education, and health promotion. Despite the promise of coaching in other fields, its use to promote the implementation of evidence-based practices, particularly within healthcare organizations, is just emerging and remains poorly understood. Recent research suggests that organizational coaching can foster change effectively by providing support and guidance that organizations require during the process of change [1–4].

Numerous models of coaching for organizational change have been developed, drawing on a variety of theories and techniques from clinical psychology, organizational development, and social psychology [5–10]. Some coaching models focus on the problem an organization is facing as a starting point for deciding how to proceed. However, more recent coaching models used in evidence-based practice implementation projects are solution-focused rather than problem-focused [5, 6]. A focus on solutions helps people develop self-efficacy and confidence in implementing other solutions. This increases organizational capacity for making and sustaining change, even when coaching is no longer available [5]. Many popular coaching models also use techniques found in cognitive-behavioral therapy to help teams reflect on organizational barriers and explore possible steps to mitigate them [7–10].

While specific coaching techniques can vary by model, there are many commonalities in how coaches interact with their coachees. Most coaches aim to facilitate critique of and reflection on current practices, as doing so often provides the best opportunities for growth in organizations. However, one study found that only 1/3 of coaching interactions were coded as this type of interaction [11]. More generally, many studies have found that much of coaching focuses on the relationship between coach and coachee and a significant portion of the work that occurs is around the building of this relationship [12, 13]. Once this relationship is established, coaches are more able to push and challenge coachees to make changes to their practices and develop new competencies, as they have already established a strong, trusting relationship [14]. Throughout all types of coaching work, the importance of being dynamic and able to adjust according to the needs of the coachee cannot be understated, as coaches need to be flexible to best support their clients [12].

### NIATx Coaching

NIATx (formerly the Network for Improvement of Addiction Treatment) is a manualized approach to promoting performance improvement and the implementation of evidence-informed innovations in organizations. The approach includes forming a change team, providing education on change strategies, and coaching sessions designed to apply tools, assess progress, and address barriers [15–17]. Like many other models of process improvement, it applies principles from social psychology [18], organizational learning [19], and process engineering [20] to foster implementation. NIATx coaching has been shown to accomplish organizational change in healthcare organizations effectively [1].

Coaches who have experience leading change projects within their own organizations and have received additional training in organizational change coaching can facilitate the NIATx process [15]. Most coaches have clinical and/or administrative backgrounds in healthcare settings. Their professional experience and previous work on change projects in their own organizations allow them to assist teams in identifying barriers to change and generating ideas to overcome these barriers.

Previous research has sought to better understand coaching, both in terms of process and outcomes. This work has focused on the importance of the working alliance between coach and coachee and on coachee characteristics. However, much remains unknown about what occurs during coaching interactions. As new coaching models are developed and applied to implementation, the need to open the “black box” grows [21–25]. Likewise, though NIATx has been established for some time, the actual work of NIATx coaching has not been examined. A better understanding of the “black box” of NIATx coaching is vital to determining which coaching elements are critical to achieving positive implementation outcomes.

In an effort to understand more about the “black box” of NIATx coaching specifically, this paper first describes the elements of NIATx coaching, then explores the proportions and patterns of the types of coaching work delineated in the model, in order to test the model’s descriptive utility and its potential to scaffold a quantitative strategy to assess the structure and process of coaching interventions. We expected that the work that occurred during this coaching intervention would closely follow the developed “NIATx model of coaching for implementation,” but sought to verify whether this hypothesis was correct by comparing the conceptualized model to the work that occurred during an actual coaching intervention.

## Methods

### Step 1: development of a NIATx model of coaching for implementation

To develop a model of coaching for implementation, semi-structured interviews were conducted with nine NIATx coaches who have served as coaches in previous implementation projects conducted by the Center for Health Enhancement Systems Studies (CHESS) at the University of Wisconsin –Madison. The nine coaches were selected to be interviewed because of their high levels of involvement in NIATx coaching projects over the last ten years. All individuals who were asked to participate agreed. The participating coaches had backgrounds in healthcare, as department leads, administrators, mental health counselors, therapists, clinical supervisors, and executive directors. All coaches interviewed had a bachelor’s degree and more than half held a higher degree.

Coaches were contacted by email to ask if they would participate. They were then interviewed via Zoom by a member of the research team (NJ) using an interview guide composed of open-ended questions that asked the coaches to reflect on the arc of a coaching intervention. Probes and follow-up questions were used to elicit details about and examples of specific tools and techniques used. Interviews lasted between 55 and 73 min, with a mean length of 61 min. Interviews were audio-recorded and transcribed verbatim.

Transcripts were uploaded into 2020 NVivo qualitative coding software, then inductively coded by one research team member. First pass coding focused on identifying the phases of a coaching engagement and types of work conducted during these phases. Following this initial pass, the research team met to discuss the coding, leading to refinements to the initial codes, such as collapsing the codes describing the phases of a coaching engagement into broader categories, and developing several new codes–for example, codes that identified the conditions under which coaching engagements were or were not successful, which were applied in subsequent coding passes. Coding continued until the team agreed that informational redundancy had been achieved. In the final stage of the analysis, the team used memos and iterative visual displays to integrate the analytic categories.

### Step 2: exploration of the model

#### A trial to increase uptake of medication for Opiod Use Disorder (MOUD)

To develop a deeper understanding of what occurs during a NIATx coaching engagement, we analyzed forty coaching calls conducted during an intervention to test whether NIATx can be used to improve uptake of MOUD in speciality SUD treatment agencies and behavioral health clinics. Organizations participating in the study were struggling to implement MOUD, so the intervention sought to provide support and strategies to improve uptake of MOUD. Seventy-five unique clinical sites in Wisconsin, Ohio, and Florida participated in the study. Sites in the intervention arm received monthly coaching from NIATx coaches from May 2017-April 2019. Coaching calls focused on a variety of topics, which shifted over the course of the intervention. They always served as a time for clinics to provide updates on their progress in increasing use of MOUD in their clinics, share challenges they were experiencing, and collaborate with other organizations and coaches to problem solve and generate new solutions to test. Three coaches participated in the project, each assigned to coach organizations in only one of the states participating in the study [26]. While sites in both arms of the study increased their use of buprenorphine during the study period, those in the coaching arm more than doubled their buprenorphine capacity (as assessed by available buprenorphine slots) and buprenorphine use. However, there was no notable change in injectable naltrexone use in either arm [26].

### Data collection

Coaching calls were completed monthly with change teams at organizations in Wisconsin, Ohio, and Florida between May 2017-April 2019. All organizations participating in Ohio completed coaching calls together for the duration of the intervention. In Florida, coaching calls in the first half of the study were conducted with organizations in the northern and central parts of the state separately, but these groups were combined in the second half of the study, and all organizations in the state then received coaching on the same calls. Coaching calls in Wisconsin were conducted separately for organizations in the north and south of the state throughout the entire study. For all of these calls, typical participants included agency administrators and the coach assigned to that state, with a small number of clinicians also in attendance. The same group of participants attended the calls each month. All calls were recorded with consent of the participants. Approximately the same number of coaching calls from the beginning (months 1–3), middle (months 13–18), and end (months 24–28) of the intervention were analyzed. A total of 10 calls from Ohio, 13 from Florida, and 17 from Wisconsin were analyzed. To prepare for analysis, each coaching call was transcribed verbatim, and participants in the call were deidentified.

Coaching calls were an average length of 44 min (range: 12–92 min). Some calls were organized as webinars on different relevant topics with only a short portion devoted to coaching, but the majority were purely coaching. There was no protocol that coaches were asked to follow but an agenda was sent out to coaches and participants before each call to guide the conversation and this was the same across all states. Most coaches typically began the conversation with small talk and casual conversations before diving into the more substantial work of coaching, starting with a brief educational focus area followed by updates from call participants on their progress. Because there was not a set protocol for exactly how or when coaches covered these areas, coaches could talk about different topics at different times according to the needs of their organizations and were able to adjust the pace of their work to ensure that organizations were getting the support that they required.

### Data analysis

**Fig. 1:**
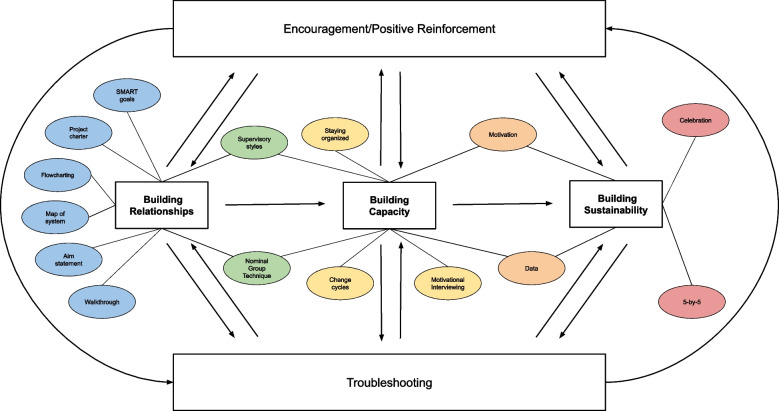
A Model of NIATx Coaching for Implementation. Colored elements are coaching tools used during each phase of the coaching process

Interview transcripts were uploaded into NVivo 2020 qualitative coding software to be analyzed. Coding was completed using a directed content analysis using a priori thematic codes based on a NIATx model of coaching for implementation (Fig. 1). Codes covered the main types of coaching work as laid out in this model (Table 1). The coding framework was developed by two research team members (KF, NJ) and was revised through discussion with other members of the research team (JH, TM). Three members of the research team completed the coding, two of whom had no involvement in the development of the coding scheme. Typically only “coach talk” was coded, as the focus of this research is on the work of the coach, but coachees’ talk was included when needed to contextualize the work that the coaches were doing. All coders first coded the same transcript to ensure appropriate overlap and establish intercoder reliability. Differences were discussed, and intercoder reliability of greater than 82% for all categories was achieved. Coders coded the remainder of the transcripts independently, with each coder focusing on coaching calls from only one state, but communicated regularly to discuss questions about the coding. Coding concerns were also discussed with the larger research team at regular intervals.

**Table 1:** Descriptions and examples of codes used. Further explanations of the codes and their basis in the model of coaching for implementation will be presented in the results

Code	Description	Example
Building relationships	asking questions, listening actively to change team members, being open to organization’s ideas, viewing the project’s aim through the lens of the change team, listening actively and empathetically	“I am really looking forward to hearing how things evolve in the updates. Feel free to use me on these calls or via email or text in the future off cycle if you have questions or concerns that come up or if you are looking for maybe quick access to a resource or you are heading into a meeting or you have got something coming up tomorrow and you are usually able to lay your hands on something. That can be helpful in this kind of engagement work that we are deep into right now.”
Building capacity	Provide education and accountability, giving examples, keeping change teams organized and on task with their changes	“I have the quickstart roadmap in front of me that may have been filled out by people in the room during our time together last month. I’m really interested in where you went from there and if you changed your thinking at all about what your goals are and what steps you’ve taken, and then we’ll have a discussion about that.“
Building sustainability	Validating work change teams have done, celebrating successes, encouraging continued work	“we’re just going to dig into that a little as a group, and think… so keep thinking about who you ask to do what, in terms of, um, in terms of the… how a person is admitted to treatment with a medication, how their ongoing treatment is sustained and managed, um, and we’ll be working through some of that together.”
Providing positive reinforcement	Celebrating successes big and small, validating challenges that are occurring	“it sounds like you are, and you have four new patients which is really the purpose of this particular process improvement series is trying to figure out how to expand capacity, and both you and your staff and [redacted]’s staff have been able to do that which is absolutely great”
Troubleshooting	Suggesting ways to overcome challenges, strategizing solutions	“You know one of the things I would just suggest around workflow is use a flowchart. Literally draw it out. So when you are trying to talk with people about changing workflow, one of the advantages of having the flow chart is you can show them where the not so great parts are and get their ideas around that.”
Tools	Rapid change cycles, nominal group technique, flowchart, 5-by-5	“if you’re still working towards this walk in ability, have you guys put together any sort of process flow diagram as to how walk ins would work?”

Percentage coverage of different codes was analyzed by the timeframe of the coaching call and by state. NVivo outputs for each file coded were utilized and percentages were averaged across files to allow comparison according to variables of interest. The team ran comparisons between files from the same state and from the same timeframe in NVivo to ensure consistency between coders, similarity of the coaching process across coaches participating in the study, and similarity of coaching occurring at each timeframe across states.

## Results

### A model of NIATx coaching for implementation

Based on interviews with experienced NIATx coaches, the research team at the University of Wisconsin – Madison described a model of coaching for implementation to understand what occurs during coaching interventions (Fig. 1). Three main types of work occur during an intervention: building relationships, building capacity, and building sustainability.

### Building relationships

The early stages of a coaching intervention are typically devoted to building relationships that allow for trust and open communication between the coach and the members of the organization participating in the coaching. Many coaches identified the relationship with their organizations as the most important predictor of success in a coaching intervention, so this work is vital to coaching success. Important relationships are primarily those between the NIATx coach and the organizational change team but can also include interactions with members of senior management and other organizational leadership.

In this first phase of coaching, coaches use various tools and skills to build strong relationships with their change teams. They seek to understand the dynamics and skills of the organization, learn more about the challenges that the organization is facing, and establish the aims and goals of the organization during this part of the coaching intervention. Consequently, coaches must ask questions, listen actively to the change team members, and be open to the organization’s experiences and ideas. Coaches need to be able to view the project and its aim through the lens of the change team by listening actively and empathetically.

### Building capacity

The second phase of coaching focuses on building the organization’s capacity, so they have the resources, support, skills, and knowledge needed to successfully address the change(s) identified in phase one. The relationships developed during the first phase allow the coaches to build momentum in their projects and facilitate trying new ways of working, even if the organization is uncomfortable or unsure how to proceed. However, relationship building remains important and continues during this phase of coaching. The focus then shifts toward putting in place the structures and processes needed for the organization to make its proposed changes.

During this phase, a large part of the coach’s role is to provide education and accountability to the organization around their proposed changes. Preferred educational methods include giving examples of steps others have taken in similar past projects and providing structure around actions to take and new approaches to try. Staying organized is especially vital during this phase of coaching. Organizations may be working on many projects at once and need assistance staying focused on their planned changes and accountable for what they said they would do. Coaches help change teams stay on task by sending summary emails after each meeting outlining planned next steps and goals before the next meeting and by encouraging teams to keep their project charters updated.

### Building sustainability

In the third phase of coaching, focus shifts to creating the conditions necessary to sustain the changes that an organization has made. For many coaches and organizations, this is the most challenging part of a coaching intervention. An important part of sustainability is validating the work a change team has put into making successful changes, as continued willingness to work on changes often stems from satisfaction with previous efforts. However, this phase of coaching is often the least developed and tends to occur following the coaching intervention.

The three main phases of coaching intervention are not entirely discreet, and work done in different phases often overlaps. Progress through a coaching intervention may occur in a relatively linear fashion, moving from phase one to phase two to phase three. However, it is not uncommon for coaches to revisit previous phases. For this reason, flexibility and adaptability are vital skills, allowing a coach to identify the need to shift focus and if a team needs to revisit a previous stage at any point during their coaching intervention.

### Other components of coaching for implementation

Outside of the main phases discussed, several overarching types of work occur during a coaching intervention. The foundation of most coaching interventions is troubleshooting, as coaches spend much of their time understanding the organization’s problems and figuring out steps forward. After change teams have begun implementing changes and challenges start to arise with implementation, coaches guide the troubleshooting that needs to occur to mitigate these problems. While the organizations are likely to know what is causing the problems, it can be difficult for them to identify solutions by themselves. Having an experienced coach to assist in identifying possible solutions can be vital to promoting successful implementation.

The other overarching element is providing positive reinforcement and encouragement, which is used to ease frustrations, support continued intervention, and promote sustainability of changes. Because not every change is successful, it can be frustrating to continue working when projects are not going as expected. Coaches provide positive reinforcement for successful changes and continued encouragement for working on larger goals of the organization. The coach’s endorsement of the positive impacts of a change project helps teams to feel supported and to recognize the impact of the changes that they have made so far, even if they feel that their effort was insufficient. Support from a coach motivates a team to continue working on changes without being impeded by frustration.

An element of the model not represented in Fig. 1 is how much direction the coach provides over the course of an intervention. Ideally, coaches provide more direction in the early stages of their coaching and less as the project continues, until change teams are working largely independently with minimal support from their coach. However, because a team’s need for support often fluctuates over a coaching intervention, the amount of coach direction also fluctuates. This again emphasizes the need for coaches to be flexible and adaptable so that they help the change team to develop the independence needed to adopt and sustain changes while also providing the support needed to diffuse frustration.

In general, coaches described coaching interventions as proceeding fairly linearly through these types of work. Still, significant flexibility exists to return to earlier types of work or skip ahead as needed according to the organization’s needs and in reality, coaches often discussed needing to return to earlier phases multiples times throughout the intervention. Troubleshooting and providing positive reinforcement occur throughout coaching interventions. Coaches employ a variety of NIATx tools, using rapid change cycles frequently. While this general structure is common across coaches, the ultimate execution of coaching can vary based on the project aims, the organization’s needs, and the coach’s background.

#### Application of the model

**Fig. 2:**
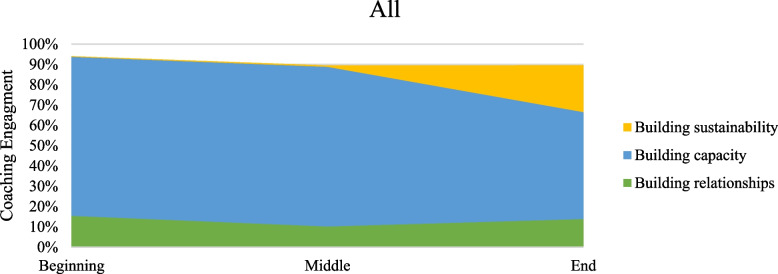
Percentage of main coaching processes occurring across all states across time periods

**Fig. 3:**
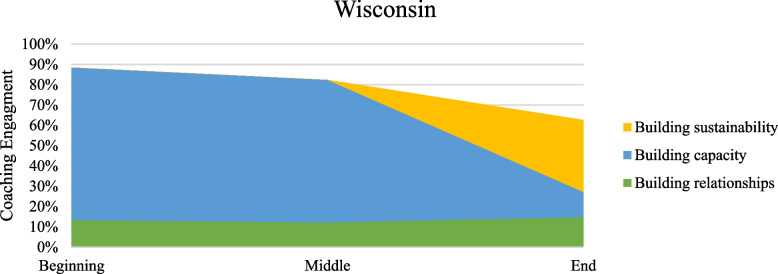
Percentage of main coaching processes occurring in Wisconsin across time periods

**Fig. 4:**
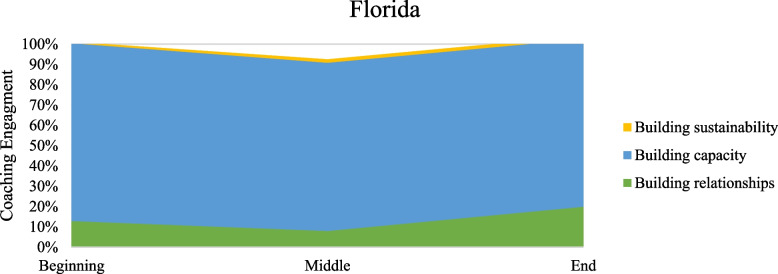
Percentage of main coaching processes occurring in Florida across time periods

**Fig. 5:**
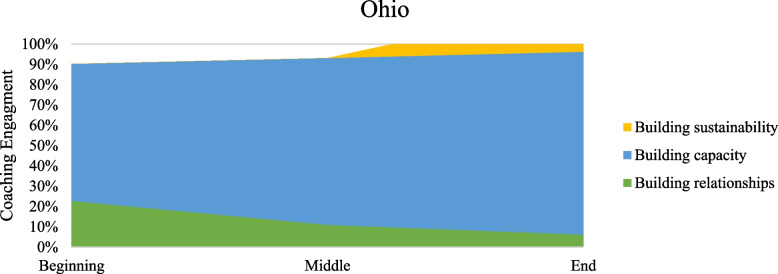
Percentage of main coaching processes occurring in Ohio across time periods

Table 2 presents percent coverage for the types of work described in the model of coaching for implementation as performed in the coaching intervention of increasing use of buprenorphine. Percentages were calculated by dividing the passage coverage coded as that behavior by the total passage coverage. More than one coaching task could be assigned to a particular passage when coaching passages. Figures 2, 3, 4 and 5 present graphs showing proportional representation of the three main types of coaching work occurring (building relationships, building capacity, and building sustainability) during the coaching engagements analyzed. Figure 2 shows percentages averaged across all states, while Figs. 3, 4, and 5 show the percentages for Wisconsin, Florida, and Ohio, respectively.

**Table 2:** Percentage of call spent on each coaching task by state and timeframe

Coaching Task	Overall	WI	FL	OH	Beginning	Middle	End
Building relationships	13.50%	13.53%	12.91%	14.29%	15.45%	10.20%	13.90%
Building capacity	71.17%	55.32%	85.08%	78.58%	78.33%	78.63%	52.64%
Building sustainability	6.80%	12.27%	1.15%	6.92%	0.27%	0.65%	23.06%
Troubleshooting	14.96%	19.03%	12.19%	13.06%	15.81%	17.71%	10.85%
Providing positive reinforcement	7.95%	11.11%	4.70%	8.01%	8.24%	6.51%	8.97%
Tools	8.54%	12.34%	5.31%	7.63%	8.55%	8.85%	8.19%
Directiveness (Amount of Coach talking)	42.51%	35.07%	47.68%	44.94%	45.79%	40.71%	39.95%

Across states and time periods, the bulk of the coaching intervention was devoted to building capacity through the development of technical skills. While the percentage of the coaching call devoted to building capacity did fluctuate depending on the coaching time period, this was the most frequent work occurring throughout the entirety of the coaching intervention. Coaches devoted similar amounts of work to building capacity at the beginning and middle periods of a coaching intervention, but less work at the end. Building relationships was emphasized most heavily at the beginning of the intervention. However, the focus on building relationships continued throughout the coaching intervention. Although there was some attention focused on building sustainability, especially at the end of the coaching intervention, this type of coaching work was seen the least in our data. Even during the end period of the coaching intervention, across all states coaches devoted less than 25% of the calls to building sustainability.

Troubleshooting and providing positive reinforcement occurred across all time periods of the coaching intervention. The percentage of work devoted to each of these processes was similar across states and coaching time periods. Coaches did nearly twice as much troubleshooting as they did providing positive reinforcement. The use of NIATx tools also stayed relatively constant across all time periods and states. Although directiveness is hard to measure directly, the percentage of time in a coaching call when the coach is speaking versus when the coachees are talking served as a proxy in our data. A pattern of decreasing directiveness was seen, with the percentage of time coaches spent talking decreasing throughout the intervention and the percentage of time coachees spent talking increasing. Finally, differences between states were seen during some phases of the coaching intervention. A smaller amount of work was devoted to building capacity in Wisconsin than in either of the other states. A very small amount of work was spent building sustainability in Florida compared to the others.

## Discussion

The coaching that occurred during the intervention aligned relatively closely with what the NIATx model of coaching for implementation (Fig. 1) would predict. Coaches focused more on building relationships in the earlier parts of the intervention and paid more attention to building capacity in the middle parts of the intervention across all states. Troubleshooting and providing positive reinforcement occurred over the course of the entire coaching intervention, as did the use of coaching tools. The directiveness of the coaches also seemed to decrease over the course of the intervention. All these observations aligned well with what would be expected based on this model of coaching for implementation.

However, some aspects of this coaching intervention differed from what the model of coaching for implementation would lead us to expect. Relationship building was emphasized most heavily at the beginning and end of the coaching intervention, with a drop-off in the middle. One potential reason for the increase in relationship building during the last portion of coaching was the turnover in change team personnel and executive leadership experienced by sites over the two-year course of the intervention. However, prior research suggests that the relationship is the most important aspect of coaching work, as stronger coach-coachee relationships have promoted better coaching outcomes, so this result may not be surprising [12, 22, 24]. This may point to a need to reevalute the relative importance placed on building the relationship throughout our model, as it may be understated currently.

The very low levels of sustainability building in the end period of the intervention also did not align well with the model. We would have expected coaches to devote the same amount of work to building sustainability at the end of the intervention as they had on building relationships in the beginning and building capacity in the middle. It is clear that organizations are not discussing ways to maintain changes they are making during their coaching sessions and that coaches fail to promote sustainability in their coaching as much as the amount of sustainability building was extremely low.

The emphasis on building capacity throughout the intervention and across states suggests that this is the main work of coaching and is prioritized by coaches. The technical skill-building that occurs during the capacity building coaching phase is vital to making organizational changes. This focus aligns well with the goals of the NIATx model coaching for implementation. Additionally, because building capacity can be accomplished in many different ways and can be a lengthy process, and because so many of the NIATx tools have this as their aim, it makes sense that the majority of a coaching intervention is devoted to this task.

However, the significant work put into capacity building cannot serve its purpose long-term without developing a greater understanding of how to build sustainability. Overall, the work devoted to building sustainability was extremely low. While coaches emphasized the importance of this type of coaching work when interviewed, it is clear it is not happening during coaching interventions. There seems to be a gap between the understanding of the need to promote sustainability and the tools available to do so. In many coaching interventions, the need to build capacity never really ends as there are always more improvements to be made and further developments that can occur. It can be difficult to shift from devoting attention to developing these changes because the improvements are always ongoing. Building sustainabilty is an important area to develop further because changes made during a coaching intervention are only useful if organizations can sustain them after coaching has ended. Finding tools and techniques that allow organizations to sustain improvements will be vital to creating more effective changes.

It is clear from this analysis that while there are distinct categories of work occurring within the model, that coaching for implementation is much less linear and phased than our model initially posited. The importance of building relationships is high throughout the entirety of the coaching intervention, not just in the beginning of the work, and coaches placed a strong emphasis on building capacity throughout the entirety of the intervention as well. This finding aligns well with prior research that shows that coaching tends to occur more in loops than in a linear manner [27].

Finally, this model achieved its goal of creating a strategy to quantitatively assess what occurs during a coaching intervention and measure the different types of work occurring, by allowing us to quantify and compare how coaches spend their time during a coaching intervention. In the future, being able to tie these quantitative measures to outcome measures will allow coaches to refine their coaching methods to ensure that they most effectively achieve organizations’ implementation goals.

### Limitations

This analysis was based on a single coaching project and analyzed a select number of coaching calls. Therefore, information from this analysis may be limited and not generalizable. Coaching calls from each state and multiple time periods were analyzed to develop a comprehensive view of the coaching process and increase its generalizability.

Additionally, there may be some differences in coding across states because of the use of different coders. While all coders received the same training and intercoder reliability was established by dual coding a call transcript and discussing coding issues as needed throughout the coding period, there may still be some differences in the ways that different coders analyzed the coaching calls. However, some differences may also be attributable to different circumstances within organizations and states and differences in coaching styles.

Using percentages to compare and contrast the type of work occurring at different times during the coaching process creates a general outline of what occurs during a coaching project, but it is not a perfect way to do so. The percentages are primarily based on when the coaches were speaking in these transcripts, but it was sometimes necessary to also include when the coachees were speaking to contextualize the work that was occurring. A more rigorous lexical analysis would allow for a more nuanced understanding of coach/coachee interactions, but would be difficult to scale for quantitative assessment. Our approach, while less sophisticated, is more easily applied. This may somewhat limit the internal validity of these findings.

Additionally, our method of coding primarily when the coaches were speaking may have elided some of the nuances of coaching work. Because coaching conversations must be constructed between two parties, assessing in this way may have left out important details and context provided by coachees [21, 23, 24, 28]. Our analysis of coach directiveness, specifically, may be weakened by being based on the proxy measurement of the amount of talking that coaches did. Future research may want to code all conversants in order to better understand how both parties contribute to the coaching process.

### Implications

We hope that this model will serve as a starting point for both coaches and researchers to develop a deeper understanding of the coaching for implementation process. Applying the coding structure we developed to look at how coaches engage with their coachees may allow coaches an objective means to assess and improve their work. For example, coaches in this project believed that they were promoting sustainability, but coaching call analysis showed that very little sustainabiltity work actually took place. Deeper understanding also will allow researchers to refine the strategy of using coaching for implementation. While our research design did not allow us to measure whether specific elements of coaching for implementation lead to improved outcomes in a coaching intervention, we hope that future work will use this model and our assessment strategy to link patterns of coaching processes to implementation coaching outcomes.

## Conclusion

The work that occurred during this coaching intervention aligned closely with the model of NIATx coaching for implementation we have described, although we also found areas where coaching diverged from the model. Our results suggest the utility of this model, but more testing will be required. Future research should focus on linking specific proportions and patterns of coaching processes to coaching outcomes to improve the efficiency and effectiveness of coaching interventions.

## Data Availability

Anonymized coaching transcripts are available upon request to the authors.
